# An effective active surveillance method for controlling nosocomial MRSA transmission in a Japanese hospital

**DOI:** 10.1007/s10156-013-0584-y

**Published:** 2013-04-05

**Authors:** Daisuke Ohkushi, Yuki Uehara, Akira Iwamoto, Shigeki Misawa, Shigemi Kondo, Kenichiro Shimizu, Satoshi Hori, Keiichi Hiramatsu

**Affiliations:** 1 Department of Infection Control Science, Juntendo University Faculty of Medicine, 2-1-1 Hongo, Bunkyo-ku, Tokyo, 113-8421 Japan; 2 Department of General Medicine, Juntendo University School of Medicine, Tokyo, Japan; 3 Department of Clinical Laboratory, Juntendo University Hospital, Tokyo, Japan; 4 Department of Clinical Laboratory Medicine, Juntendo University School of Medicine, Tokyo, Japan

**Keywords:** Methicillin-resistant *Staphylococcus aureus*, Active surveillance, Horizontal transmission

## Abstract

Hospital-wide active surveillance for methicillin-resistant *Staphylococcus aureus* (MRSA) targeted to adult patients with a history of MRSA carriage within the past 5 years was performed in Juntendo University Hospital (JUH) over a 2-year period. In the first year, MRSA screening culture was ordered by physicians in charge. In the second year, infection-control practitioners (ICPs) took samples for active surveillance culture. The average monthly transmission rate of MRSA in JUH was 0.35 per 1,000 bed-days in the first year and decreased significantly to 0.26 per 1,000 bed-days in the second year (*P* < 0.05). In the second year, more active commitment of ICPs to MRSA screening was effective in improving the performance rate of screening, shortening turn-around time of screening results, and decreasing transmission rate. Increasing compliance with active MRSA surveillance by involvement of ICPs, targeting patients with a previous history of MRSA carriage in the previous 5 years, was effective to control nosocomial MRSA transmission.

## Introduction

Since its discovery in 1961 [1], methicillin-resistant *Staphylococcus aureus* (MRSA) has remained a major nosocomial pathogen throughout the world, causing grave clinical and financial problems in healthcare facilities [2]. Implementation of contact precautions for MRSA carriers is essential for preventing its nosocomial spread, so early detection of newly hospitalized patients carrying MRSA is a critical issue [3]. Active surveillance culture (AS-C) for MRSA has been conducted in Juntendo University Hospital (JUH) since 2006 for hospitalized patients having a history of MRSA carriage in the past 5 years. While waiting for the results of screening culture, healthcare workers (HCWs) followed a pre-emptive contact precaution policy, but the efficacy of this policy has not been evaluated. We postulated that a shorter turnaround time (TAT) of screening results could be expected by more active involvement of infection-control practitioners (ICPs) and shorter TAT might cause higher compliance to implement the contact precaution policy and to reduce nosocomial transmission of MRSA.

 In this study, we analyzed the effect of our active surveillance practice on the rate of horizontal MRSA transmission in the hospital, especially of the commitment of ICPs to active MRSA screening.

## Patients and methods

### Study setting and periods

JUH is a large, 1,020-bed teaching hospital with an average hospital stay of 13.2 days. All adult wards (922 beds) were included in this study. The study periods were divided into two phases: January–December 2009 (phase 1), and January–December 2010 (phase 2).

### Candidates for active surveillance of MRSA and screening procedures

AS-C was conducted in both phases of the study. All patients with a history of MRSA carriage in the last 5 years were candidates for AS-C on admission. The following patients were excluded: (1) those who had history of confirmed MRSA carriage/infection within 14 days before admission (regarded as MRSA carriers on admission); (2) those previously assigned as MRSA carriers but who had at least three successive negative culture results from anterior nares and other body sites on three separate days before admission (regarded as ex-carriers); and (3) patients who declined to participate in the study (regarded as MRSA carrier throughout their hospitalization). Electronic medical records of patients with a past history of MRSA carriage were flagged automatically on admission. In phase 1, nasal culture for MRSA screening was ordered by physicians in charge when they recognized that their new patients had a history of MRSA carriage. For patients admitted at nights or on holidays, physicians in charge ordered screening culture on earliest business days after their admission.

In phase 2, ICPs identified candidates for AS-C from electronic medical records of scheduled admission cases 1 day before admission. On admission, ICPs immediately visited inpatients’ rooms to obtain nasal swabs. In cases of urgent admissions or admissions on holidays, patients were screened by ICPs on the day after their admission. Specimens were taken from patients’ anterior nares using sterile cotton swabs moistened with saline. The swab sample was streaked onto MRSA screening agar (CHROMagar™ MRSA; CHROMagar Microbiology, Paris, France) [4] and incubated at 35 °C for 48 h. When colonies were found on screening agar, the patient was regarded as a MRSA carrier. The culture results were returned to HCWs in charge via electronic hospital medical charts.

### Infection-control policies, isolation, and compliance monitoring

The usual contact precaution policy was implemented immediately after admission of all candidates for AS-C while awaiting culture results. Isolation in single rooms was encouraged if they were available, and the patients agreed with isolation. Contact precaution and isolation policy was continued if the first culture on admission revealed the presence of MRSA or the candidate did not participate in AS-C. When the first culture was negative, two additional cultures of anterior nares and cultures from another body sites where MRSA had been positive previously were taken by physicians in charge. If all additional cultures were negative, screening candidates were released from isolation. Average days from admission to confirmation of culture results were calculated as TAT in phase 1 and phase 2. Use of alcohol hand rub (AHR) was monitored monthly throughout all adult wards in phase 1 and phase 2 as an indirect indicator of compliance with hand-hygiene procedures for preventing MRSA nosocomial transmission. In order to examine the effect of AHR on the transmission rate, the entire study period was also divided into four subphases: phases 1a (6 months from January to June 2009), 1b (6 months from July to December 2009), 2a (5 months from January to May 2010), and 2b (7 months from June to December 2010).

### MRSA transmission rate

An event of MRSA transmission was defined as a positive culture obtained for the first time later than 48 h after admission. The monthly transmission rate was calculated as the number of MRSA transmission events in all adult wards, divided by the number of bed-days. The outcome was calculated as the average monthly transmission rate and expressed as events per 1,000 bed-days.

### Statistical analysis

Data analysis was performed with the Mann–Whitney *U* test or the chi-square test using Excel (Microsoft Corporation, USA). Values of *P* < 0.05 were considered as significant difference.

### Ethical disclosure

This study was approved by the ethical committee of JUH with the approval number 21–84. Written informed consent was obtained from all participants.

## Results

### Implementation of active surveillance culture

Table 1 shows the results of implementation of AS-C in phases 1 and 2. A total of 239 patients were enrolled for AS (1.12 % of 21,399 annual admissions) in phase 1 and 255 (1.16 % of 22,070 annual admissions) in phase 2. However, AS-C was performed on only 179 (74.9 % of 239) patients enrolled in phase 1, and execution of AS-C increased significantly to 235 (92.2 % of 255) in phase 2 (*P* < 0.01). Percentage of MRSA-positive patients in all patients who underwent AS-C in phase 1 was 38.0 % (68 of 179) and increased significantly to 54.5 % (128 of 235) in phase 2, respectively (*P* < 0.01). Average TAT was 3.45 days in phase 1 and improved significantly to 2.97 days in phase 2 (*P* < 0.001, Table 1).

**Table 1:** Comparison of parameters in phases 1 and 2

Comparison individuals	Phase 1	Phase 2	*P* value
No. of patients admitted in all adult wards in each phase	21,399	22,070	–
No. of candidates for screening (% of total admitted patients)	239 (1.12 %)	255 (1.16 %)	0.705
Active surveillance culture (AS-C)			
Percentage of eligible patients screened in candidates for screening	74.9 %	92.2 %	<0.001
No. of MRSA-positive patients (% in all patients who underwent AS-C)	68 (38.0 %)	128 (54.5 %)	<0.001
Average turnaround time (TAT) from admission (mean ± SD)	3.45 ± 1.48 days	2.97 ± 1.18 days	<0.001
Average monthly consumption of alcohol hand rubs (mean ± SD)	10,308 ± 1,411 ml	12,894 ± 2,627 ml	<0.05
Percentage of candidates allocated to single-room isolation on admission	64.0 %	71.0 %	0.098
Monthly transmission rate of MRSA (mean ± SD, per 1,000 patient days)	0.35 ± 0.16	0.26 ± 0.08	<0.05

*MRSA* methicillin-resistant *Staphylococcus aureus*,* SD* standard deviation,* AS-C* active surveillance culture

### MRSA transmission rate and related factors

The average monthly transmission rate of MRSA was 0.35 (0.079–0.62) per 1,000 bed-days in phase 1 and decreased significantly to 0.26 (0.078–0.38) in phase 2 (*P* < 0.05, Table 1). The average monthly consumption of AHR also significantly increased in phase 2 (12,894 ml) compared with phase 1 (10,308 ml) (*P* < 0.05, Table 1).

**Fig. 1:**
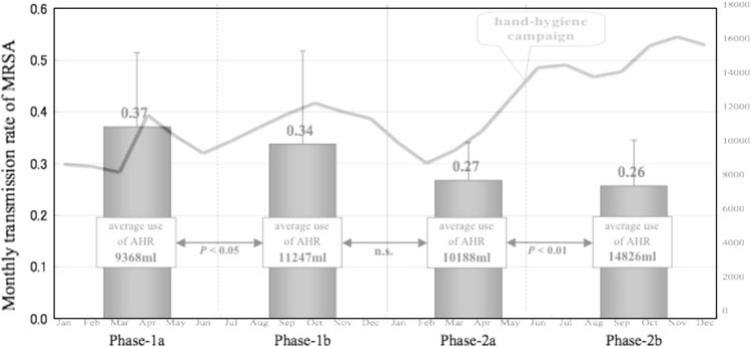
Comparison of average monthly transmission rate of methicillin-resistant *Staphylococcus aureus* (MRSA) per 1,000 bed-days and average monthly consumption of alcohol hand rubs (AHRs) between four subphases. *Bar graphs* show average monthly transmission rate of MRSA per 1,000 bed-days. A *line graph* shows total consumption of AHR per month in adult wards of Juntendo University Hospital. The transmission rates in each subphase were not statistically different. Average consumption of AHR was significantly different between phases 1a and 1b (*P* < 0.05) and between phase 2a and 2b (*P* < 0.01)

Comparison of MRSA nosocomial transmission rates between each intervention subphase and the change of AHR consumption is shown in Fig. 1. The average MRSA transmission rate gradually decreased throughout the entire study period, although the transmission rates in each subphase were not statistically different. This tendency was not in accord with the average consumption of AHR.

The number of candidates allocated to single-room isolation on hospital admission was 153 in phase 1 and 181 in phase 2. The percentage of candidates who were isolated appropriately showed no statistically significant difference in different phases of the study: 153/239 in phase 1 (64.0 %) versus 181/255 in phase 2 (71.0 %) (*P* = 0.098, Table 1).

## Discussion

This study was conducted to investigate the effectiveness of an active screening culture policy to reduce MRSA transmission rate in JUH. A retrospective review of MRSA carriers hospitalized in 2005, a year with no surveillance culture and isolation policy, showed that 67 of 69 carriers (97.1 %) had history of MRSA carriage or infection within the past 5 years (Hori, unpublished data). From this result, we decided to target patients who had episodes of MRSA carriage or infection within the past 5 years as candidates for AS-C instead of indicating universal screening for all admissions.

The rate of MRSA transmission significantly decreased during phase 2 of this study. Between the two phases, four other important differences were observed: the larger number of at-risk patients screened; the higher rate of detection of MRSA-positive patients; the shorter TAT of culture results; and the higher consumption rate of AHR. These differences, except for the increase in AHR use, seem most likely to be due to involvement of ICPs to the first AS-C. ICPs were able to identify enrolled patients prior to admission and performed subsequent sampling during phase 2. Immediate visit and sampling by ICPs after admission contributed to the lower omission rate of screening, and the number of screened patients increased in phase 2. Adequate and standardized procedures to obtain culture samples and shorter time from sampling to beginning laboratory incubation are generally important to increase positivity culture rates. Therefore, sampling by ICPs and shorter TAT may have contributed to the higher rate of MRSA-positive patients in phase 2, although it is possible that more MRSA carriers were included by chance in phase 2; also, screened patients could not be released from isolation until two additional culture results were negative. In addition, ICP visits may have encouraged HCWs in charge to more strictly implement infection-control procedures after patient admission.

In May 2010, a project to encourage AHR use was conducted in the hospital by giving awards to wards that demonstrated excellent compliance with the policy. During this project, the use of AHR rose from 10,188 to 14,826 ml (*P* < 0.01); however, the monthly MRSA transmission rates did not significantly change (0.27 vs. 0.26 per 1,000 bed-days, *P* > 0.05). Monitoring total AHR consumption is considered one of the indicators to measure compliance of hand hygiene procedure in hospitals [5], and this study raised the possibility that improved compliance with hand hygiene was one cause for the decrease in MRSA transmission rate. However, correlation between AHR consumption and MRSA transmission rate was unclear in comparison with shorter subphases in this study. Other factors besides infection control for MRSA could increase the use of AHR, so the effect of increasing AHR consumption needs to be investigated in the future. Similarly, single-room isolation on admission did not seem to contribute to a decrease in MRSA transmission between phases 1 and 2 of this study, so other factors appear to be involved.

The efficiency of AS-C depends on the prevalence of MRSA carriers. Although complete surveillance would potentially detect all carriers, universal screening of all hospitalized patients is not practical in terms of cost effectiveness [2, 6]. A significant merit of our screening policy is that it is conducted on only about 1 % of patients admitted and yet has a significant impact on MRSA transmission rate in the hospital. MRSA carriage rates among patients who underwent AS-C were as high as 38.0 and 54.5 % in phase 1 and 2 studies (Table 1). In hospital-wide universal screening, the prevalence of MRSA among patients on admission was reported as only 2.7–7.5 % [7–9]. Therefore, our screening policy was highly effective in detecting MRSA carriers despite the small number of patients screened.

A limitation of this study was that the correct horizontal transmission rate in the hospital could not be analyzed, as we neither did universal screening covering all hospital wards nor had all MRSA strains isolated in this hospital. Cases transferred from other hospitals were excluded as candidates for AS-C in this study but clearly represent a risk factor for MRSA import to JUH.

In conclusion, active MRSA surveillance targeting of patients with a history of MRSA carriage within the previous 5 years was highly effective in detecting MRSA carriers on admission. Increasing compliance with screening these high-risk patients by active involvement of ICPs was effective in controlling nosocomial MRSA transmission.
